# Occupational Stress and Cardiovascular Risk Factors in High-Ranking Government Officials and Office Workers

**DOI:** 10.5812/ircmj.11747

**Published:** 2014-08-01

**Authors:** Seyyed Jalil Mirmohammadi, Mahmoud Taheri, Amir Houshang Mehrparvar, Mohammad Heydari, Ali Saadati Kanafi, Mehrdad Mostaghaci

**Affiliations:** 1 Department of Occupational Medicine, Shahid Sadoughi University of Medical Sciences, Yazd, IR Iran; 2 Department of Surgery, Tehran University of Medical Sciences, Tehran, IR Iran

**Keywords:** Cardiovascular Risk Factor, Burnout, Professional, Managers

## Abstract

**Background:**

Cardiovascular diseases are among the most important sources of mortality and morbidity, and have a high disease burden. There are some major well-known risk factors, which contribute to the development of these diseases. Occupational stress is caused due to imbalance between job demands and individual’s ability, and it has been implicated as an etiology for cardiovascular diseases.

**Objectives:**

This study was conducted to evaluate the cardiovascular risk factors and different dimensions of occupational stress in high-ranking government officials, comparing an age and sex-matched group of office workers with them.

**Patients and Methods:**

We invited 90 high-ranking officials who managed the main governmental offices in a city, and 90 age and sex-matched office workers. The subjects were required to fill the occupational role questionnaire (Osipow) which evaluated their personal and medical history as well as occupational stress. Then, we performed physical examination and laboratory tests to check for cardiovascular risk factors. Finally, the frequency of cardiovascular risk factors and occupational stress of two groups were compared.

**Results:**

High-ranking officials in our study had less work experience in their current jobs and smoked fewer pack-years of cigarette, but they had higher waist and hip circumference, higher triglyceride level, more stress from role overload and responsibility, and higher total stress score. Our group of office workers had more occupational stress because of role ambiguity and insufficiency, but their overall job stress was less than officials.

**Conclusions:**

The officials have higher scores in some dimensions of occupational stress and higher overall stress score. Some cardiovascular risk factors were also more frequent in managers.

## 1. Background

It has been predicted that cardiovascular diseases, notably atherosclerosis will have the most total disease burden in the world by the year 2020. Ischemic heart diseases are the leading causes of mortality and morbidity in many developed countries. There are two types of cardiovascular risk factors: those which are modifiable by changing lifestyle or drug therapy, and those which are fixed, such as age and sex. Major risk factors of atherosclerosis include cigarette smoking, hypertension, low high-density lipoprotein (HDL) cholesterol, diabetes mellitus, positive family history (of premature coronary heart disease), older age, obesity, physical inactivity, and poor diet. Thus, to reduce the mortality and morbidity of ischemic heart diseases, we must try to control these risk factors (1).

Occupational stress is defined as a perceived imbalance between job demands and the individual’s ability to work, when the failure leads to important consequences. A large number of studies have implicated workplace stressors as the etiology of coronary heart disease (2-4). Common workplace stressors include organizational stressors (e.g. change, inadequate communication and inter-personal conflict), concerns about career development (e.g. lack of promotional opportunity or unemployment), role stressors (e.g. role conflict and role ambiguity), task stressors (e.g. quantitative and qualitative overload or underload, and responsibility for the lives and well-being of others), and work environment stressors (e.g. poor aesthetics, physical exposures, noise, and odors) (2).

The overall findings of studies about job stress suggest that two main job stressors identified in the Karasek et al. model (5) play a significant role: work demands and job control, although there are some exceptions (6) and methodological issues exist regarding how demands and control measures should be defined and measured (7). Some work practices such as shift work and working overtime, as well as more general psychosocial factors like reduced job complexity, social support, and job insecurity are considered to be associated with increased risk of cardiovascular heart diseases. It has been known for a long time that some degree of arousal and activation is necessary to reach appropriate levels of performance (8). A study which compared managers to other employees has shown higher demands, higher levels of conflict, and low degree of social support from peers in managers, but they had higher scores in the psychosocial work environment factors such as job satisfaction, perceived management quality from their managers, influence, degrees of freedom at work, possibilities for development and meaning of work (9). Another study compared job stress in managers, professionals, and clerical personnel, and the study has shown that managers had job pressures more than professionals, while ‘lack of opportunity for advancement’ and ‘inadequate salary’ were the most prominent stressors of the clerical workers (10). Serious health consequences may result when job stress becomes too severe or persists for a long time (11).

Cardiovascular risk factors and occupational stressors have been studied extensively in different groups of occupations (4, 9, 10). Managerial jobs seem to be prone to some types of occupational stressors due to its obligations and requirements. There have been studies, which evaluated cardiovascular risk factors in occupations, which included managerial work with high responsibilities, and these studies have had different, sometimes inconsistent results (12, 13).

## 2. Objectives

This study was designed to evaluate occupational stress and cardiovascular risk factors in high-level government officials, and to compare those with the same variables in office workers with no managerial tasks.

## 3. Patients and Methods

Yazd is the capital city of a province in Iran, with population of more than 400000 people. There are studies, which have shown a high prevalence of cardiovascular events among high-ranking officials (14). Also, there were some cardiovascular events among the members of this occupational group in this province; so in a case-control study in 2012, we evaluated 90 high-ranking government officials who were compared with a group of age and sex-matched office workers regarding their cardiovascular risk factors and occupational stress. The sample size was measured by the following formula considering the frequency of occupational stress among high-ranking officials to be 1.5 times the ordinary office workers (9), with the power of 80%:

First, we prepared a list of main government offices in Yazd. This list included the contact information of about 300 main government offices and their chief administrators. Of these, 98 offices were chosen (generally biggest offices with the most number of employees, or those with a higher order of precedence, e.g. the governor’s and the mayor’s offices, the military offices, and the major universities). Top managers of each office and a sex and age-matched ordinary office worker were invited for the study. An invitation letter was sent to each of these 98 offices, requesting the chief manager (or principal) of the office to refer to the occupational medicine clinic. These people formed our “government officials” group. This group included the highest ranking authorities of the city. In the invitation letter, we asked the officials to refer with at least 10 hours of fasting, and also bring one office worker from the same office with the same sex and age (± 2 years). These subjects formed our second group: office workers. We compared most of the evaluated risk factors and occupational stress categories between these two groups, who had the same sex, age, and work environments, but different job characteristics.

When the participants attended the clinic, they received a questionnaire in which they were asked about their birth date, occupational history (their current organization and job title, work experience in the current job, and whether they have a second job or not), past medical history (diabetes mellitus, hypertension, hyperlipidemia, or ischemic heart disease), history of smoking (number of cigarettes smoked per day, and years of smoking, which then were multiplied to calculate pack-years of cigarette smoking), family history of premature coronary heart disease (defined as the onset of coronary heart disease before 55 years in subject’s male first-degree relatives, or before 65 years in subject’s female first-degree relatives) (1).

After answering these questions, the subjects were required to answer an occupational stress questionnaire. We used a Persian translation of the occupational role questionnaire (Osipow and Spokane) (15), which is a five-point Likert type questionnaire with 60 questions. All items are self-report format. The answers to these questions range from “rarely or never true” to “true most of the time.” The answers to these questions got scored from 1 to 5. Therefore, each scale of 10 questions was related to one dimension of occupational stress and provided a score of 10-50. The effect of each stressor is categorized into four grades (low: 10-19, low to moderate: 20-29, moderate to severe: 30-39, severe: 40-50):

- Role overload (RO) measures the extent to which job demands exceed resources (personal and workplace), and the extent to which an individual is able to accomplish expected workloads.
-Role insufficiency (RI) measures the extent to which the individual’s training, education skills and experience are appropriate to job requirements.
- Role ambiguity (RA) measures the extent to which the priorities, expectations, and evaluation criteria are clear to the individual.
- Role boundary (RB) measures the extent to which an individual is experiencing conflicting role demands and loyalties in the work setting.
- Responsibility (R) measures the extent to which an individual has or feels a great deal to responsibility for the performance and welfare of others on the job.
- Physical environment (PE) measures the extent to which an individual is exposed to high levels of environmental toxins or extreme physical conditions.

These scales can be used separately or summed to produce different measures of stress (16). The Persian translation of this questionnaire had been used in other studies, and in one of these studies the Cronbach α coefficient was 0.83 (17). After calculating the score for every scale of occupational stress (except for physical environment score), the sum of other 5 scales were calculated to estimate the overall tension score, which was in the range of 50-250. We disregarded the stress from physical environment in our study, because each pair of a manager and an office worker was invited from the same governmental office (mostly from the same work environment), so we expect same stress from physical environment. Before proceeding to physical exam, the questionnaire was checked to see if there are unfilled sections. If a question was left unanswered, most probably its concept would be misunderstood by the subject. Thus, the question was explained, and we requested it to be filled.

After filling the questionnaire, a physical exam was performed, and the subject’s weight, height, blood pressure, waist and hip circumference were measured. The weight was measured using a digital scale which showed body weight in kilograms with accuracy of 100 grams. The height was measured in centimeters using a standard height meter. Body mass index (BMI) was calculated as weight (kg) divided by square of height (m^2^). Blood pressure was measured in mmHg using a standard mercury sphygmomanometer. Waist circumference was assessed in centimeters using a tape measure, horizontally at the midpoint between the last rib and iliac crest, and the hip circumference was measured in centimeters using the same tape measure at the greater trochanters (18).

After the physical exam, the subjects were referred to clinic’s lab, and a venous blood sample was obtained to check for fasting glucose, total cholesterol, HDL cholesterol, and triglyceride. It was asked in the invitation letter that the participants should fast at least for 10 hours before attending the occupational clinic. If a subject had any food or drink in the past 10 hours, he was asked to refer to the clinic for the lab tests another time, when he fasted for at least 10 hours. Ultimately, of 98 offices asked to send their lead manager and an office worker, 90 offices participated in our research, giving a response rate of 91%. An informed consent was obtained from each participant.

The data from all of subjects were entered in the IBM SPSS statistics software version 19, and analyzed using chi-square, independent samples *t* tests, and Mann-Whitney U test. Kolmogorov-Smirnov test was used to test the normality of data and showed that TG and HDL were not normally distributed, so for their comparison Mann-Whitney U test was used.

In one step of our analysis, we divided the quantitative values into special subgroups, based on the information provided by up-to-date scientific references about cardiovascular risk factors (19). Our goal was to determine if working as a high-ranking official has put our subjects at different risk for being in an abnormal category of these risk factors. Fasting glucose level was divided into 3 categories: < 110 mg/dL, 111-125 mg/dL, and > 125 mg/dL. Cholesterol level was divided into 2 categories: less than 200 mg/dL, and ≥ 200 mg/dl. Triglyceride level was divided into 2 categories: 1-200 mg/dL and > 200 mg/dL. HDL level was divided into 2 categories: < 40 mg/dL and ≥ 4 0 mg/dL. BMI value was divided into 4 categories: ≤ 18.5 kg/m^2^, 18.51–25 kg/m^2^, 25.01–30 kg/m^2^, and > 30 kg/m^2^. Systolic blood pressure was divided into 3 categories: 1-119 mmHg, 120-139 mmHg, and ≥ 140 mmHg. Diastolic blood pressure was divided into 3 categories: 1-79 mmHg, 80-89 mmHg and ≥ 90 mmHg.

The study was approved by the ethics committee of Shahid Sadoughi University of Medical Sciences (number: 2432, 23.4.2011). Appropriate, relevant ethical standards (based on the declaration of Helsinki), and current best practice was adhered to conduct this study. All subjects were ensured about the privacy of their information.

## 4. Results

The study subjects were 180 employees (90 high-ranking officials and 90 office workers). The mean age of all subjects was 47.48 ± 5.39. One hundred and seventy-five subjects were males and 2 were females (age and sex were matched between the two groups). The mean work experience was 11.21 ± 9.72 in all subjects. We also calculated the pack-years of cigarette smoking in two groups, and our high-ranking officials had smoked fewer pack-years of cigarettes. The mean pack-years of smoking were 1.03 ± 4.21. Also, 19 officials (21.1%) and 20 office workers (22.2%) had a second job (P value = 1.00). Table 1 compares some demographic data between two groups. The results of the self-reported past medical history and second jobs are shown in Table 2. Table 3 summarizes the results from our measurements (the lab data and physical exams). Table 4 compares the risk factor categories between the high-ranking officials and office workers. We also analyzed different dimensions of job stress in our groups. The results of this comparison are shown in Table 5.

**Table 1.** Comparison of Demographic Data Between Two Groups ^a^

	Results	Range	P Value
**Age, y**			0.98
High-ranking officials	47.46 (5.67)	32-64	
Office workers	47.51 (5.13)	31-62	
**Work experience, y**			< 0.001 ^b^
High-ranking officials	6.47 (7.48)	2-13	
Office workers	16 (9.39)	1-30	
**Smoking (pack-year)**			0.02 ^b^
High-ranking officials	0.33 (1.99)	0-7	
Office workers	1.73 (5.57)	0-35	

^a^ Data are presented as Mean ± SD.

^b^ Mann-Whitney U-test.

**Table 2.** Self-Reported Past Medical History of Cardiovascular Risk Factors ^a, b^

	Cardiovascular Risk Factors				
	DM	HLP (Total)	Hypertension	CHD	CHD Family History
**High-ranking officials**	9 (10.0)	30 (33.3)	16 (17.7)	9 (10.0)	17 (18.8)
**Office Workers**	15 (16.6)	25 (27.7)	16 (17.7)	6 (6.7)	18 (20.2)
**P value**	0.27	0.12	1.00	0.59	0.85

^a^ Abbreviations: DM, diabetes mellitus; HLP, hyperlipidemia; CHD, coronary heart disease.

^b^ Data are presented as No. (%).

**Table 3.** Comparison of the Mean ± SD Values for Physical Examination and Lab Data Results Between the High-Ranking Officials and Office Workers ^a, b^

	Height, m	Weight, kg	BMI, kg/m2	Systolic BP, mmHg	Diastolic BP, mmHg	Waist Cir, cm	Hip Cir, cm	FG, mg/dL	Chol, mg/dL	HDL, mg/dL	TG, mg/dL
**Mean value in high-ranking officials**	1.71 ± 0.07	83.43 ± 11.52	28.41 ± 3.09	123.39 ± 14.35	81.87 ± 9.09	97.89 ± 7.77	102.9 ± 6.33	113.58 ± 34.8	196.73 ± 40.74	56.4 ± 35.33	208.71 ± 139.81
**Mean value in office workers**	1.69 ± 0.06	79.77 ± 14.3	27.51 ± 4.09	125.17 ± 13.7	81.29 ± 8.24	93.64 ± 9.72	100.67 ± 7.55	112.84 ± 36	193.36 ± 44.62	53.33 ± 9.51	169.14 ± 72.93
**P value**	0.25 ^c^	0.06 ^c^	0.097 ^c^	0.39 ^c^	0.70 ^c^	< 0.001 ^c^	0.03 ^c^	0.89 ^c^	0.60 ^c^	0.63 ^d^	0.017 ^d^

^a^ Abbreviations: BMI, body mass index; BP, blood pressure; Waist Cir, waist circumference; Hip Cir, hip circumference; FG, fasting glucose; Chol, cholesterol; HDL: high density lipoprotein; TG, triglyceride.

^b^ Data are presented as Mean ± SD.

^c^ t-test.

^d^ Mann-Whitney U-test.

**Table 4.** Comparison of the Frequency of Risk Factor Categories Between the High-Ranking Officials and Office Workers ^a, b^

	Cardiovascular Risk Factor Categories																		
	FG, mg/dL			Chol, mg/dL		TG, mg/dL		HDL, mg/dL		BMI, kg/m^2^				Systolic BP, mmHg			Diastolic BP, mmHg		
**Category**	62-110	111-125	> 125	< 200	≥ 200	53-200	> 200	< 40	≥ 40	< 18.5	18.51-25	25.01-30	> 30	100-119	120-139	≥ 140	60-79	80-89	≥ 90
**High-ranking officials**	60 (69.7)	9 (10.5)	17 (19.8)	49 (57.0)	37 (43.0)	55 (64.0)	31 (36.0)	7 (8.1)	79 (91.9)	0	12 (13.3)	56 (62.2)	22 (24.5)	30 (33.3)	47 (52.2)	13 (14.5)	32 (35.5)	42 (46.7)	16 (17.8)
**Office workers**	61 (73.5)	5 (6.0)	17 (20.5)	55 (66.3)	28 (33.7)	62 (74.7)	21 (25.3)	1 (1.2)	82 (98.8)	0	20 (22.5)	48 (53.9)	21 (23.6)	29 (32.6)	46 (51.7)	14 (15.7)	26 (29.2)	50 (56.2)	13 (14.6)
**P value**	0.57			0.26		0.13		0.06		0.25				0.97			0.44		

^a^ Abbreviations: HDL, high density lipoprotein; BMI, body mass index; BP, blood pressure; TG, triglyceride; Chol, cholesterol; FG, fasting glucose

^b^ Data are presented as No. (%).

**Table 5.** Occupational Stress in High-Ranking Officials and Office Workers

	Role Overload	Role Insufficiency	Role Ambiguity	Role Boundary	Responsibility	Total Score
**Mean stress in high-ranking officials**	32.53	24.56	20.68	24.36	33.75	135
**Mean stress in office workers**	26.82	27.60	23.17	25.49	27.19	130
**P value**	< 0.001	< 0.001	< 0.001	0.08	< 0.001	0.02

## 5. Discussion

This study was designed to evaluate the occupational stress and cardiovascular risk factors in a group of high-ranking managers, and compare them to a group of office workers. The high-ranking officials had less work experience in their current jobs and fewer pack-years of cigarette smoking, but they had higher waist and hip circumference, higher triglyceride level, more stress from role overload and responsibility and higher total stress score. Our group of office workers had more occupational stress because of role ambiguity and insufficiency, but their overall job stress was less than officials.

There are lots of studies which have implicated occupational stressors as the cause of coronary heart disease (2-4) but there are some controversies among the results of these studies. Prevention of job stress might decrease the incidence of coronary heart disease, but it seems to have a smaller effect than controlling standard risk factors like cigarette smoking (20).

Backe et al. (3) evaluated the role of psychosocial job stress for the development of cardiovascular diseases in a systematic review. They included 26 publications, describing 40 analyses out of 20 cohorts. The risk estimates for work stress increased the risk of cardiovascular disease in 13 out of the 20 cohorts. They reported that the most significant results were from analyses that only considered men, and the association between job stress and cardiovascular diseases in women were not clear (3). In the current study, there were only two women, so the results cannot be generalized to women managers. 

Malinauskiene et al. (14) studied the risk of the first-time myocardial infarction in 25 - 64 years old men with different jobs. They used the international standard classification of occupations (ISCO) categories to classify the jobs. Their research reported an increased risk of MI for the 1st ISCO category (legislators, senior officials, and managers), and they found an odds ratio of 2.18 for myocardial infarction in this category compared to 7th ISCO category (craft and related trades workers) (14). We found as well that some cardiovascular risk factors and overall occupational stress score were higher in high-ranking officials, which may contribute to future myocardial infarction attacks in this occupational group.

Gyntelberg et al. (12) assessed the job strain and cardiovascular risk factors among members of the Danish parliament, and compared them with age and sex-matched participants. The politicians group in that study reported higher job demands, but also had more influence on their job. They smoked less and were taller, but their serum lipids and blood pressure were not significantly different from those of controls (12). These results are mostly in agreement with the results of our study, since our study showed more occupational stress and fewer pack-years of cigarette smoking in high-ranking officials, and no difference was seen in systolic and diastolic blood pressure and most of the serum lipids between two groups. The high-ranking officials in our study had higher triglyceride level than the office workers, but that could be due to a few very high values of triglycerides in our officials group. When we categorized triglyceride into 2 categories (1-200 and > 200 mg/dL), there were no significant differences between the two groups (P value = 0.13).

Holme et al. (13) studied coronary risk factors in about 15000 male participants of various occupational groups aged between 40 - 49 years in Oslo. They evaluated cigarette smoking, serum cholesterol and triglyceride, systolic and diastolic blood pressure, and body weight. In addition, a “coronary risk factor (CRF)” score was calculated, using the number of cigarettes smoked per day, serum cholesterol level and systolic blood pressure for each occupational group. There were 3 occupational groups in this study involving high-ranking managers: 1) leading administrators and executive officials; 2) directors, managers, and working proprietors; 3) administrative, executive and managerial work. In this study, these three occupational groups all had lower frequency of cigarette smoking, lower triglyceride level, and systolic and diastolic blood pressure. Although the study did not include statistical measures (like P values) to investigate significant differences between the values from different groups, but overall the coronary risk factor score in these 3 groups does not seem to be drastically different from the mean CRF score (13). The results of this study are mostly in consistent with the current study.

In a systematic review by Cosgrove et al. the relationship between work-related stress and type 2 diabetes has been assessed in a review of articles until March 2010. They identified 9 studies (4 prospective, 1 case-control and 4 cross-sectional), and the meta-analyses failed to show any statistically significant associations between individual aspects of occupational psychosocial stress or job strain and risk of type 2 diabetes, which is consistent with the results of the current study (21).

Djindjic et al. (4) investigated the associations between the total occupational stress index score and hypertension, type 2 diabetes mellitus, and lipid disorders in middle-aged men and women. Their results showed that the total OSI score was associated significantly with diabetes mellitus, any type of dyslipidemia, and arterial hypertension for both genders. Their results are mostly inconsistent with our results, which were probably due to their different method of evaluating occupational stress (4).

A lot of studies have tried to evaluate occupational stress and its dimensions or psychosocial hazards in different jobs. Although there are a number of ways in which psychosocial hazards in work environment can be assessed, but the type of measures, which are formally being used are mostly self-report measures. These measures can be quite general in that they attempt to assess the general level of perceived psychosocial hazards in the workplace, or they can be quite specific and focus on one or two particular types of psychosocial hazard (11).

Turnage et al. studied 30 job stressors as measured by the job stress survey (JSS) among employees of a large manufacturing firm, including 68 managers, 171 professionals (mostly engineers), and 69 clerical personnel. Their results showed that managers experienced job pressures more than professionals/engineers, while ‘lack of opportunity for advancement’ and ‘inadequate salary’ were the most prominent stressors among clerical workers (10).

Manshor et al. (22) examined the sources of occupational stress among 440 managers working in Malaysian multinational companies. They found that workloads, working conditions and relationships at work were the managers’ main concern that may lead to stress at the workplace (22).

Skakon et al. (9) examined perceived stress and work strain among 2052 subjects (128 managers and 1924 employees) at 48 worksites. Their results showed that managers have higher demands, higher level of conflicts, and lower degree of social support than peers, but higher scores in the psychosocial work environment factors, including job satisfaction, perceived management quality from their managers, influence, degrees of freedom at work, possibilities for development and meaning of work. Overall, they suggest that managers have a lower stress level, which contradicts the general opinion that managers experience higher pressure and more stress than employees and is inconsistent with the results of the present study in which more stress was found for high-ranking officials in some stress dimensions, while office workers had more stress in some other dimensions. Our overall stress score was higher in officials’ group, which can be due to the different questionnaires which were used to assess occupational stress (9).

Nadaoka et al. (23) studied stress and psychiatric disorders in 283 local government officials in Japan, using a questionnaire consisting of a series of psychometric scales. The results of this study showed that officials in higher levels of employment were supported less and were more severely depressed than officials at lower levels of employment (23).

Our study had some limitations though. At first, we invited our participants by sending a letter to all governmental offices, explaining the research goals and asking the office principal and a matched office worker to participate in the study. Because of the busy work schedule of the government officials, some of the invited subjects were unable to attend the occupational clinic in the given time period. In these cases, we contacted their offices and discussed special appointments with them. Some of the offices did not send any office workers with their officials, or the office workers who accompanied their managers were not properly matched for the manager. We contacted special departments of these offices and asked them to choose an age and sex-matched office worker and instruct them to refer to our clinic to participate in the study. Managers or office workers of 8 governmental offices did not participate in the study. Some subjects did not fast enough for the laboratory tests, so they were asked to refer again another time for those tests. Some of these participants did not attend the laboratory again, resulting in missing data in the lab tests.

The strong points of this study include collecting a relatively large sample of high-ranking managers and a matched control for each; to the best of our knowledge, the first study in Iran on this issue.

The weak points of the study include the low number of participating females, and the lack of the possibility to retest lab data. In this study, the high-ranking managers had more overall job stress and higher score in some dimensions of occupational stress. Also some of the evaluated cardiovascular risk factors were more common in high-ranking managers, although some of the other dimensions of job stress and some cardiovascular risk factors were less common among them. The results of this study mainly agreed with most substantial and significant articles and systematic reviews with similar subjects. Overall, there is a considerable inconsistency between the results from different studies, which can mostly be due to different methods of measuring occupational stress, or different characteristics of the study populations.
